# CDK9 activity is critical for maintaining MDM4 overexpression in tumor cells

**DOI:** 10.1038/s41419-020-02971-3

**Published:** 2020-09-15

**Authors:** Monika Štětková, Kateřina Growková, Petr Fojtík, Barbora Valčíková, Veronika Palušová, Amandine Verlande, Radek Jorda, Vladimír Kryštof, Václav Hejret, Panagiotis Alexiou, Vladimír Rotrekl, Stjepan Uldrijan

**Affiliations:** 1 Faculty of Medicine, Department of Biology, Masaryk University, Kamenice 5, 625 00 Brno, Czech Republic; 2 International Clinical Research Center, St. Anne’s University Hospital, Pekařská 53, 656 91 Brno, Czech Republic; 3 Laboratory of Growth Regulators, Palacký University and Institute of Experimental Botany, The Czech Academy of Sciences, Šlechtitelů 27, 783 71 Olomouc, Czech Republic; 4 Faculty of Medicine and Dentistry, Institute of Molecular and Translational Medicine, Palacký University, Hněvotínská 5, 779 00 Olomouc, Czech Republic; 5 Central European Institute of Technology (CEITEC), Masaryk University, Kamenice 5, 625 00 Brno, Czech Republic

**Keywords:** Oncogenes, Melanoma, Mechanism of action

## Abstract

The identification of the essential role of cyclin-dependent kinases (CDKs) in the control of cell division has prompted the development of small-molecule CDK inhibitors as anticancer drugs. For many of these compounds, the precise mechanism of action in individual tumor types remains unclear as they simultaneously target different classes of CDKs – enzymes controlling the cell cycle progression as well as CDKs involved in the regulation of transcription. CDK inhibitors are also capable of activating p53 tumor suppressor in tumor cells retaining wild-type *p53* gene by modulating MDM2 levels and activity. In the current study, we link, for the first time, CDK activity to the overexpression of the *MDM4 (MDMX)* oncogene in cancer cells. Small-molecule drugs targeting the CDK9 kinase, dinaciclib, flavopiridol, roscovitine, AT-7519, SNS-032, and DRB, diminished MDM4 levels and activated p53 in A375 melanoma and MCF7 breast carcinoma cells with only a limited effect on MDM2. These results suggest that *MDM4*, rather than *MDM2*, could be the primary transcriptional target of pharmacological CDK inhibitors in the p53 pathway. CDK9 inhibitor atuveciclib downregulated MDM4 and enhanced p53 activity induced by nutlin-3a, an inhibitor of p53-MDM2 interaction, and synergized with nutlin-3a in killing A375 melanoma cells. Furthermore, we found that human pluripotent stem cell lines express significant levels of MDM4, which are also maintained by CDK9 activity. In summary, we show that CDK9 activity is essential for the maintenance of high levels of MDM4 in human cells, and drugs targeting CDK9 might restore p53 tumor suppressor function in malignancies overexpressing MDM4.

## Introduction

The proliferation of healthy cells is strictly controlled by cyclin-dependent kinases (CDKs) and their negative regulators, the CDK inhibitors (CDKIs)^1,2^. As deregulated cell proliferation is one of the hallmarks of human cancer^3^, pharmacological targeting of dysregulated CDK activity is seen as a promising approach to combat the unlimited cell proliferation associated with tumorigenesis^4,5^. Many compounds capable of inhibiting CDKs have been developed since the identification of the first small-molecule CDK inhibitor flavopiridol more than 25 years ago^6,7^. While some of the drugs are specific for CDKs driving cell cycle progression, the majority are pan-CDK inhibitors also targeting kinases participating in the regulation of transcription, such as CDK7 or CDK9^4,5^. In cancer cells, this can alter the production of essential pro-survival proteins, e.g., BCL-2 and MCL-1^8–11^. Besides, we and others have shown that in cancer cells retaining wild-type p53, the inhibition of RNA polymerase II-mediated transcription by small-molecule CDKIs can lead to non-genotoxic activation of p53-dependent transcription, potentially contributing to their cytotoxic activity^12–16^. Other reports have shown that DNA damage can synergize with CDKIs in inducing high p53 levels and activity^17,18^. The activation of p53 was, in some cases, accompanied by nuclear accumulation of p53, and inhibition of the expression of its major cellular E3 ubiquitin ligase MDM2 was suggested as the mechanism responsible for p53 activation in response to CDK inhibition^19,20^. However, a later study showed that the accumulation of p53 in response to the CDK9 inhibitor 5,5-dichloro-1-b-d-ribofuranosylbenzimidazole (DRB) was not due to diminished MDM2 levels^21^.

In malignant melanoma and several other cancers often retaining wild-type p53, the overexpression of a related protein MDM4 (also known as MDMX or HDMX) rather than MDM2 can play a critical role in blocking the p53 tumor suppressor^22–26^. The N-terminal part of MDM4 can bind p53 and inhibit its transcriptional activity independently of MDM2^27^. Also, the C-terminal RING finger domain of MDM4 can bind to the RING domain of MDM2 and contribute to its E3 ubiquitin ligase activity and p53 degradation^28–30^. Furthermore, a more recent study has shown that MDM4 can facilitate the p53-mediated expression of MDM2 under stress conditions^31^. Together, this suggests that MDM4 could serve as a relevant therapeutic target for reactivation of p53 signaling in melanoma and other cancers expressing increased levels of the protein.

Some reports suggest the anti-melanoma activity of CDKIs might be dependent on p53 signaling^32^. A high number of new small-molecule compounds targeting CDKs have been developed and characterized in recent years, many of which have already entered clinical trials^4,33^. Therefore, in the current study, we take advantage of the recently developed clinical candidates and compounds with more narrow selectivity. We revisit the issue of p53 pathway regulation in response to pharmacological modulation of CDK activity, concentrating on the previously little-appreciated role for MDM4 in the negative control of p53 in some tumor types.

## Results

### CDK inhibitors roscovitine and flavopiridol activate p53 in melanoma cells expressing high levels of MDM4

**Fig. 1:**
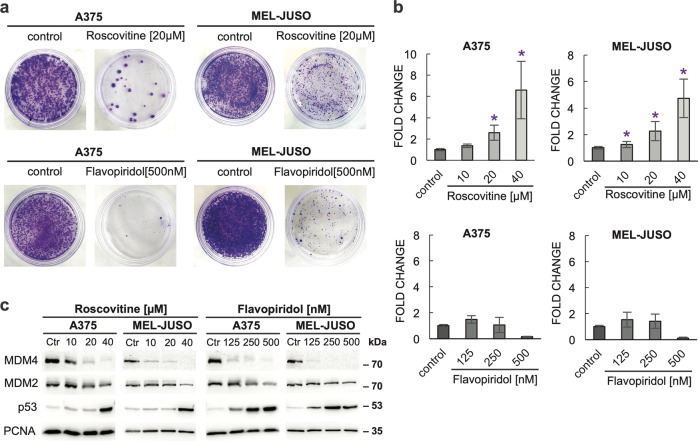
Pan-specific CDK inhibitors roscovitine and flavopiridol inhibit the growth of melanoma cell lines expressing high MDM4 levels. **a** Colony formation assay: A375 and MEL-JUSO cells were treated with 20 µM roscovitine or 500 nM flavopiridol for 24 h, washed, and further cultivated in a drug-free growth medium. Cells were fixed and stained with 6.0 % glutaraldehyde + 0.5 % crystal violet solution. **b** Activation of p53 by roscovitine and flavopiridol: A375 and MEL-JUSO cells stably transfected with a p53 transcriptional activity luciferase reporter construct were treated with the indicated concentrations of roscovitine and flavopiridol for 24 h, lysed, and luciferase activity in lysates was determined. Relative p53 transcriptional activity was calculated. The values represent the mean ± SD; *N* = 3; *P < 0.05. **c** Roscovitine and flavopiridol downregulate MDM4 at lower concentrations than MDM2, concurrent MDM4 and MDM2 downregulation leads to stabilization of p53. Western blot analysis of protein lysates of A375 and MEL-JUSO cells after 24-h treatment with CDK inhibitors. The levels of PCNA protein served as a loading control.

Human melanomas often retain wild-type p53 but express high levels of MDM4^24^. To determine the extent of p53 activation in response to pharmacological CDK inhibition, we stably transfected melanoma cell lines A375 and MEL-JUSO with pGL4.38[luc2P/p53 RE/Hygro] luciferase reporter construct and measured luciferase activity in lysates of cells treated with two CDKIs under clinical development, *R*-roscovitine (seliciclib) and flavopiridol (alvocidib). Both drugs strongly inhibited the growth of melanoma cells in vitro (Fig. 1a). In the p53 activity assay, roscovitine induced significant concentration-dependent upregulation of p53-mediated luciferase expression, while the increase of p53-dependent transcription was surprisingly small in flavopiridol-treated cells (Fig. 1b). We reasoned that the known activity of flavopiridol towards the Positive Transcription Elongation Factor, P-TEFb, might prevent the reporter expression in flavopiridol-treated cells at the concentrations used, even if active p53 levels were increased^34,35^. Therefore, in the next step, we focused on the p53 protein levels and the levels of its two primary negative regulators MDM2 and MDM4 in CDKI-treated cells using western blot. In both cell lines, we detected an increase in p53 levels in response to three different concentrations (125, 250, 500 nM) of flavopiridol, while roscovitine induced robust p53 stabilization only in the highest (40 μM) concentration tested (Fig. 1c). Surprisingly, MDM2 levels remained relatively stable, with a significant decrease observed only in cells treated with the highest CDKI concentrations. On the contrary, MDM4 protein levels dropped already in response to the lowest tested drug concentration (0.125 μM flavopiridol and 10 μM roscovitine) in both cell lines (Fig. 1c).

### Expression of MDM4, rather than MDM2, is the target of pharmacological CDK inhibitors in melanoma and breast carcinoma cells

**Fig. 2:**
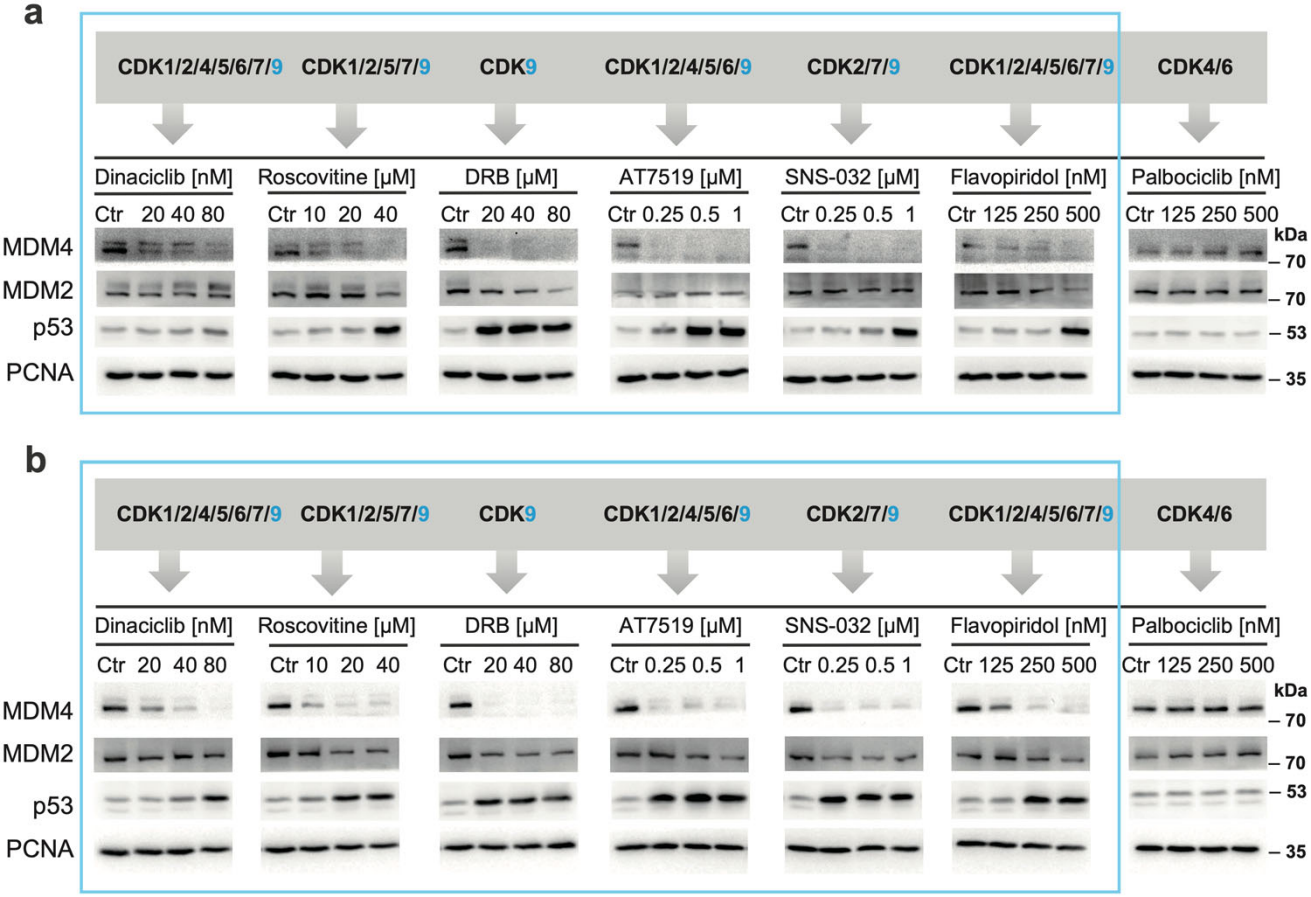
Pan-CDK inhibitors predominantly target MDM4 expression in melanoma and breast carcinoma cells. Palbociclib, lacking activity toward CDK9, affects neither MDM4 nor MDM2 levels. Western blot analysis of cancer cell lysates after 24-h treatment with the indicated concentrations of CDK inhibitors in A375 (**a**) and MCF7 cells (**b**).

As our initial results suggested that the *MDM4* expression in cancer cells might be extremely sensitive to pharmacological CDK inhibition, significantly more than the expression of *MDM2*, we decided to test the inhibitory potential of a more extensive panel of commonly used CDKIs towards MDM4 and MDM2 levels in cancer cells. The response of malignant melanoma A375 (Fig. 2a) and MCF7 breast carcinoma cells (Fig. 2b), both overexpressing MDM4, was determined using western blotting. Twenty-four-hour treatments with CDK9/Cyclin T (P-TEFb) inhibitors DRB and flavopiridol, as well as pan-specific CDKIs roscovitine, dinaciclib (SCH727965), AT-7519, and SNS-032 (BMS-387032), all caused a sharp dose-dependent drop in MDM4 levels in both cell lines while having only limited effect on MDM2 protein that was in some cases observed only at the highest tested concentrations. Interestingly, even a small decrease in MDM2 expression was commonly accompanied by a significant p53 stabilization. In contrast, a CDK4/CDK6-specific compound palbociclib (PD0332991) exhibiting no activity towards CDK9 did not induce changes in MDM4/MDM2 protein levels (Fig. 2a, b). To confirm that the selected concentrations of the CDK4/CDK6 inhibitor affected cell cycle progression, using flow cytometry, we measured the incorporation of the modified thymidine analog EdU into newly synthesized DNA in controls treated with DMSO and cells treated with palbociclib. Two CDKIs also used at sub-micromolar concentrations, dinaciclib and flavopiridol, were included for comparison. Data presented in Fig. S1a confirmed a significant impact of palbociclib on cell cycle progression. As expected, CDKIs dinaciclib and flavopiridol, capable of arresting cells in all phases of the cell cycle, were more efficient in inhibiting DNA synthesis than palbociclib that selectively inhibits progression through the G1 phase^4,36^.

### CDK9 inhibition/depletion diminishes MDM4 expression in melanoma cells

**Fig. 3:**
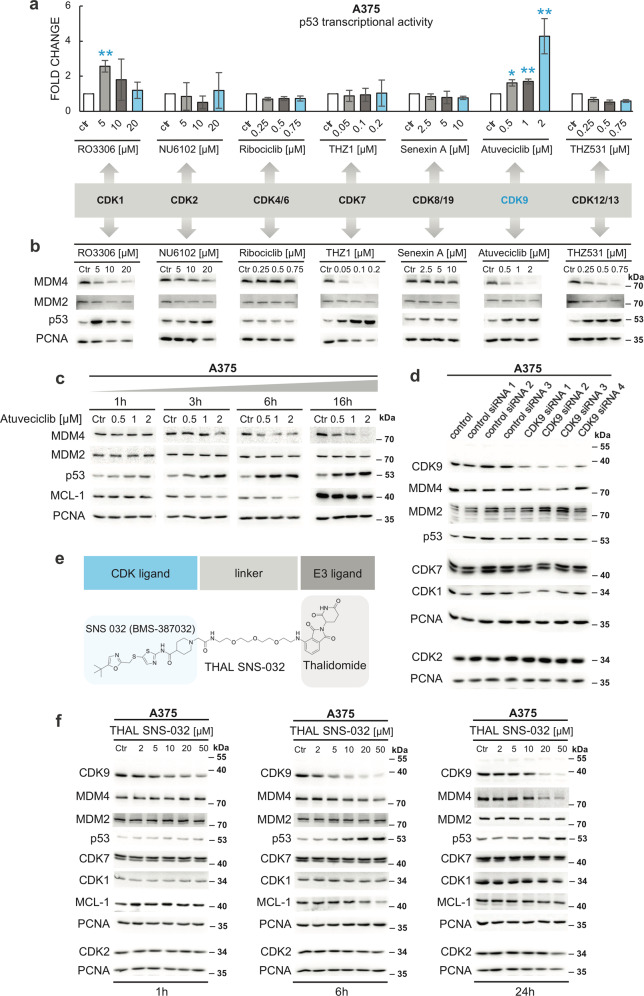
CDK9 inhibition diminishes MDM4 expression and activates p53 in melanoma cells. **a** Activation of p53-dependent transcription by CDK9 inhibitor atuveciclib and CDK1 inhibitor RO3306. A375 cells expressing luciferase under the control of a p53-responsive promoter were treated with specific CDK inhibitors for 24 h. Relative p53 activity in cell lysates was determined. The values represent the mean ± SD; *N* = 3; *P < 0.05; **P < 0.01. **b** Inhibitors of transcriptional CDKs THZ1, THZ531, and atuveciclib inhibit MDM4 expression and stabilize p53, with minimal impact on MDM2 levels. Western blot analysis of A375 cell lysates after 24-h treatment with the indicated concentrations of specific CDK inhibitors. **c** Time-course experiment with CDK9 inhibitor atuveciclib. A375 cells were treated with atuveciclib for 1, 3, 6, and 16 h. Proteins of interest were visualized by western blotting. **d** CDK9 knockdown downregulates MDM4 levels. Western blot analysis of A375 response to siRNA-mediated CDK9 knockdown. Commercially available non-targeting siRNAs (control siRNA 1) and GFP-targeting siRNAs (control siRNA 2 and 3) were used as controls. **e** CDK9 PROTAC THAL-SNS-032. The dual affinity molecule binds E3 ubiquitin ligase CRBN and induces selective degradation of the CDK9 protein. **f** Selective knockdown of CDK9 by THAL-SNS-032 induces a decrease in MDM4 levels. Western blot analysis of A375 cell lysates after 1, 6, and 24-h treatment with the indicated concentrations of THAL-SNS-032.

Many ATP-competitive pan-specific CDKIs can bind ATP pockets of other kinases. Therefore, in the next experiment, we analyzed the impact of a different set of highly selective CDK inhibiting compounds on the p53 pathway in A375 cells. The set included molecules preferentially targeting a small subset of CDKs: RO3306 (CDK1), NU6102 (CDK2), ribociclib (CDK4/6), THZ1 (CDK7), senexin A (CDK8), atuveciclib (BAY1143572; CDK9), and THZ531 (CDK12/13)^33,37^. When we analyzed the transcriptional activity of p53 in A375 cells using the luciferase reporter cell line, a significant upregulation of p53 activity was detected only in cells treated with the CDK9 inhibitor atuveciclib and, surprisingly, the CDK1 inhibitor RO3306 (Fig. 3a). Considering the limitations of the luciferase reporter system when assessing p53-dependent transcription in cells treated with compounds potentially negatively affecting transcription, we analyzed the changes in the levels of p53 and its negative regulators MDM2 and MDM4 also directly, using western blotting (Fig. 3b). While ribociclib and senexin A did not induce any significant changes, inhibitors of transcriptional CDKs THZ1, THZ531, and atuveciclib all potently stabilized p53 and inhibited MDM4 expression, with a much weaker impact on MDM2 levels. Some effect on MDM4 was also observed with the CDK2 inhibitor NU6102, and inhibition of MDM4 expression was also present in cells treated with the CDK1 inhibitor RO3306 (Fig. 3b, Fig. S1b).

In addition to the regulation of transcription, CDK12 and CDK13 were also reported to modulate alternative splicing of some transcripts, possibly by interactions with RNA processing factors^38,39^. Interestingly, alternative splicing of the *MDM4* transcript has been reported as the primary mechanism responsible for MDM4 overexpression in cancer^40^. THZ531 could, therefore, impact *MDM4* expression by disrupting the alternative splicing. However, there is also a report of physical interaction between CDK12 and CDK9^41^. The CDK12/13 inhibitor might, therefore, act also indirectly, through affecting CDK12-CDK9 crosstalk. Moreover, as CDK7 can directly phosphorylate and activate CDK9, the effect of the CDK7 inhibitor THZ1 on MDM4 could also be mediated through CDK9^42^.

Therefore, in the following experiments, we decided to concentrate on the effects of CDK9 inhibition. We started by performing a time-course experiment with the CDK9 inhibitor atuveciclib. MDM4 downregulation was apparent after six hours in atuveciclib-treated A375 cells (Fig. 3c). Moreover, in the highest atuveciclib concentration (2 μM), a mild decrease of MDM4 could be detected already after three hours. Interestingly, p53 stabilization was observed despite no change in the levels of MDM2 (Fig. 3c). Next, we used RNA interference (RNAi) to confirm the role of CDK9 in the control of MDM4 expression. A375 cells were transfected with three different negative controls and four commercially available siRNAs targeting the *CDK9* transcript. All *CDK9* siRNAs induced a partial knockdown of CDK9 expression. Two siRNAs with the most substantial impact on CDK9 expression (CDK9 siRNA 2 and 3) also promoted a decrease in MDM4 levels without inhibition of MDM2 expression (Fig. 3d).

The PROTAC (PROteolysis TArgeting Chimera) technology can serve as an alternative to RNAi for the rapid and reversible depletion of a protein of interest in living cells^43^. We treated A375 cells with a PROTAC compound THAL-SNS-032 (Fig. 3e), a chimera between thalidomide and the CDK inhibitor SNS-032, that has been reported to promote selective degradation of CDK9 mediated by the ubiquitin ligase CRBN^44^. The compound induced nearly complete CDK9 depletion at concentrations higher than 20 μM, accompanied by a significant drop in MDM4 levels (Fig. 3f). Again, the effect on MDM2 was minimal, confirming the differential dependence of MDM4 and MDM2 protein levels on CDK9. Interestingly, the effect of THAL-SNS-032 on the expression of MDM4 was more potent than on the expression of a negative regulator of apoptosis MCL-1 (Fig. 3f), a well-established CDKI target^9,10^. A partial recovery of the CDK9 levels at 24 h could be caused by the compound instability, as previously suggested for another thalidomide-based chimeric protein degrader^45^.

### CDK9 inhibition disrupts *MDM4* gene transcription and promotes p53-dependent transcription

**Fig. 4:**
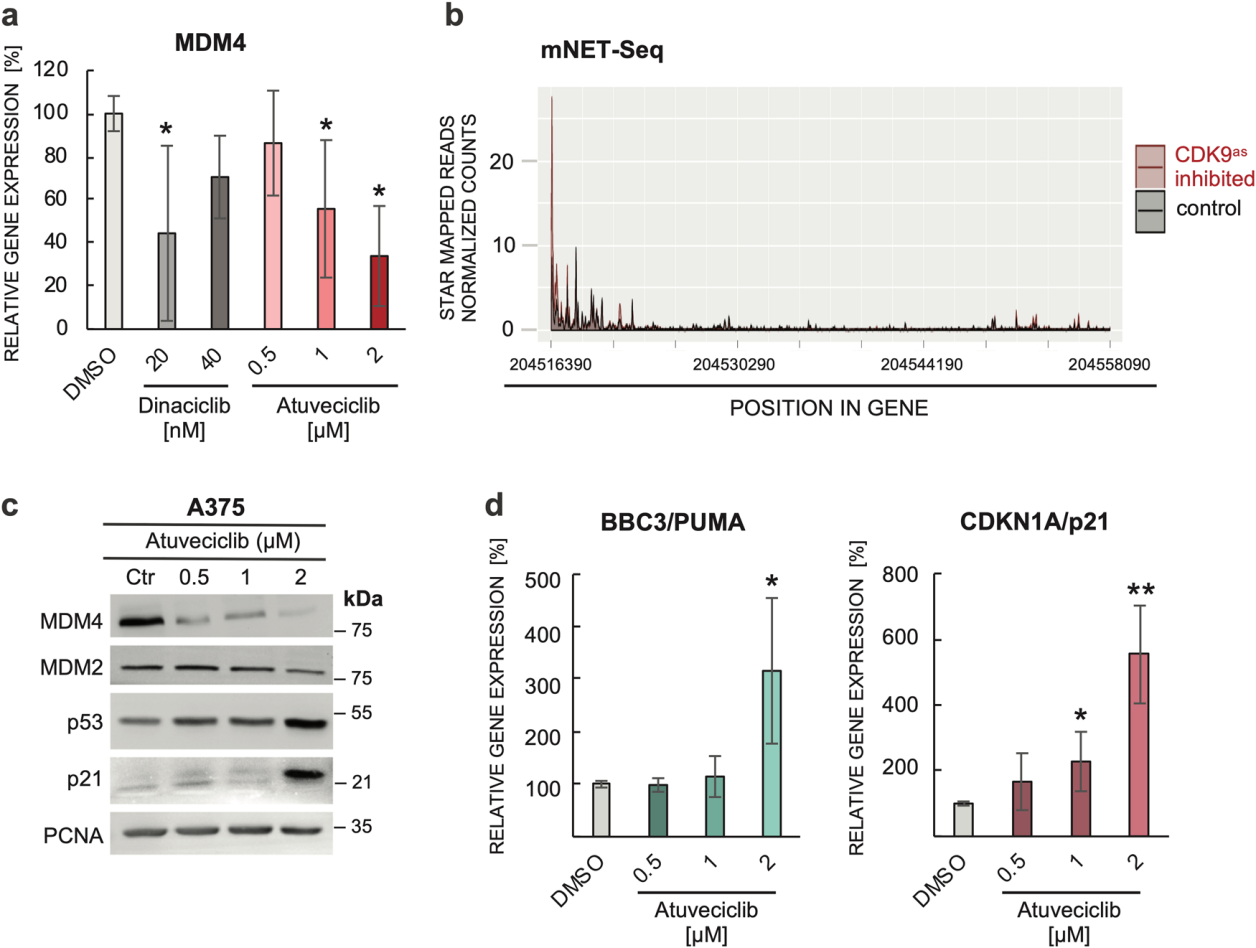
Inhibition of P-TEFb-mediated transcription contributes to MDM4 depletion. **a** *MDM4* gene expression analysis by qRT-PCR. Six-hour treatment with dinaciclib and atuveciclib was used to inhibit CDK9-dependent transcription in A375 cells. Total RNA was isolated using RNA Blue reagent, reverse transcribed, and real-time quantitative PCR was performed in triplicates. The values represent the mean ± SD; *N* = 4; *P < 0.05. **b** mNET-Seq analysis of RNA polymerase II position along the *MDM4* gene. Raji B cells harboring an analog sensitive CDK9 mutation were treated with adenine analog 1-NA-PP1 (CDK9as inhibited) or DMSO (control), and RNA polymerase II occupancy within the *MDM4* gene region between control and CDK9-inhibited cells was compared. **c** Western blot analysis of MDM4, MDM2, p53, and p21 protein levels in A375 cell lysates after 24-hour treatment with the indicated concentrations of CDK9 inhibitor atuveciclib. PCNA served as a loading control. **d** Expression analysis of p53 target genes *p21* and *PUMA* by qRT-PCR. A375 cells were treated with CDK9 inhibitor atuveciclib for 14 h before harvesting. Total RNA was isolated using RNA Blue reagent, reverse transcribed, and real-time quantitative PCR was performed in triplicates. The values represent the mean ± SD; *N* = 3; *P < 0.05; **P < 0.01.

CDK9 serves as a catalytic subunit of P-TEFb that is required for productive elongation of transcripts synthesized by RNA polymerase II^42,46^. Therefore, small-molecule CDK9 inhibitors such as DRB and flavopiridol can inhibit the elongation step of transcription^34,47^. We used real-time quantitative PCR to determine the impact of dinaciclib and atuveciclib on *MDM4* transcription in A375 cells. Both CDKIs caused a decrease in *MDM4* expression (Fig. 4a), suggesting that the inhibition of P-TEFb-mediated transcription could contribute to the observed depletion of MDM4 in CDKI-treated cells.

In a recent study, Gressel et al.^48^ analyzed the role of CDK9 in the dynamic regulation of transcription using a chemical biology approach that circumvents the off-target effects of standard chemical CDK9 inhibitors. The researchers mutated *CDK9* in the human Raji B cell line to create CDK9 protein sensitivity to the adenine analog 1-NA-PP1, which does not affect wild-type cells. This mutation then allowed for rapid and highly specific inhibition of CDK9. The results showed that CDK9 stimulates the release of paused polymerase and activates transcription by increasing the number of transcribing polymerases and thus the amount of mRNA synthesized per time^48^. Among other data generated in this study, the mNET-seq data map the RNA 3′-end of engaged RNA polymerase II and show the position of paused polymerases. We reused this data set to determine the effect of the specific CDK9 inhibition on *MDM4* gene transcription. Results presented in Fig. 4b show the position of RNA polymerase II along the *MDM4* gene in control DMSO-treated cells and cells treated with 1-NA-PP1 for 10 min to inhibit CDK9. The observed rapid increase in the proportion of polymerases occupying the 5′ portion of the *MDM4* gene indicates that CDK9 inhibition might indeed have a significant impact on *MDM4* expression.

MDM4/MDMX overexpression has been reported to serve as a critical inhibitor of p53 function in a significant proportion of human melanomas^24^, including our model cell line A375^40^. Therefore, we hypothesized that the decrease in MDM4 levels in response to CDK9 inhibition could contribute to p53 activation by CDKIs. To test it, we transfected A375 cells bearing the pGL4.38[luc2P/p53 RE/Hygro] reporter construct with siRNAs targeting *MDM4* transcripts or control siRNAs and determined the luciferase activity. In parallel, we ran western blots to determine the efficacy of MDM4 knockdown in individual samples. Results presented in Fig. S1c suggests that MDM4 indeed participates in the control of p53 activity in A375 melanoma.

In the next set of experiments, we analyzed the expression of established endogenous p53 transcriptional targets in A375 cells treated with the CDK9 inhibitor atuveciclib. Western blot analysis showed significant upregulation of the CDKN1A/p21 protein levels in response to 24-h atuveciclib treatment (Fig. 4c). Analysis of the expression using real-time quantitative PCR confirmed *CDKN1A/p21* upregulation also on the mRNA level. The analysis also showed an increased expression of another p53 target *BBC3/PUMA* in atuveciclib-treated cells (Fig. 4d).

### CDK9 inhibitor atuveciclib synergizes with MDM2 inhibitor nutlin-3a

**Fig. 5:**
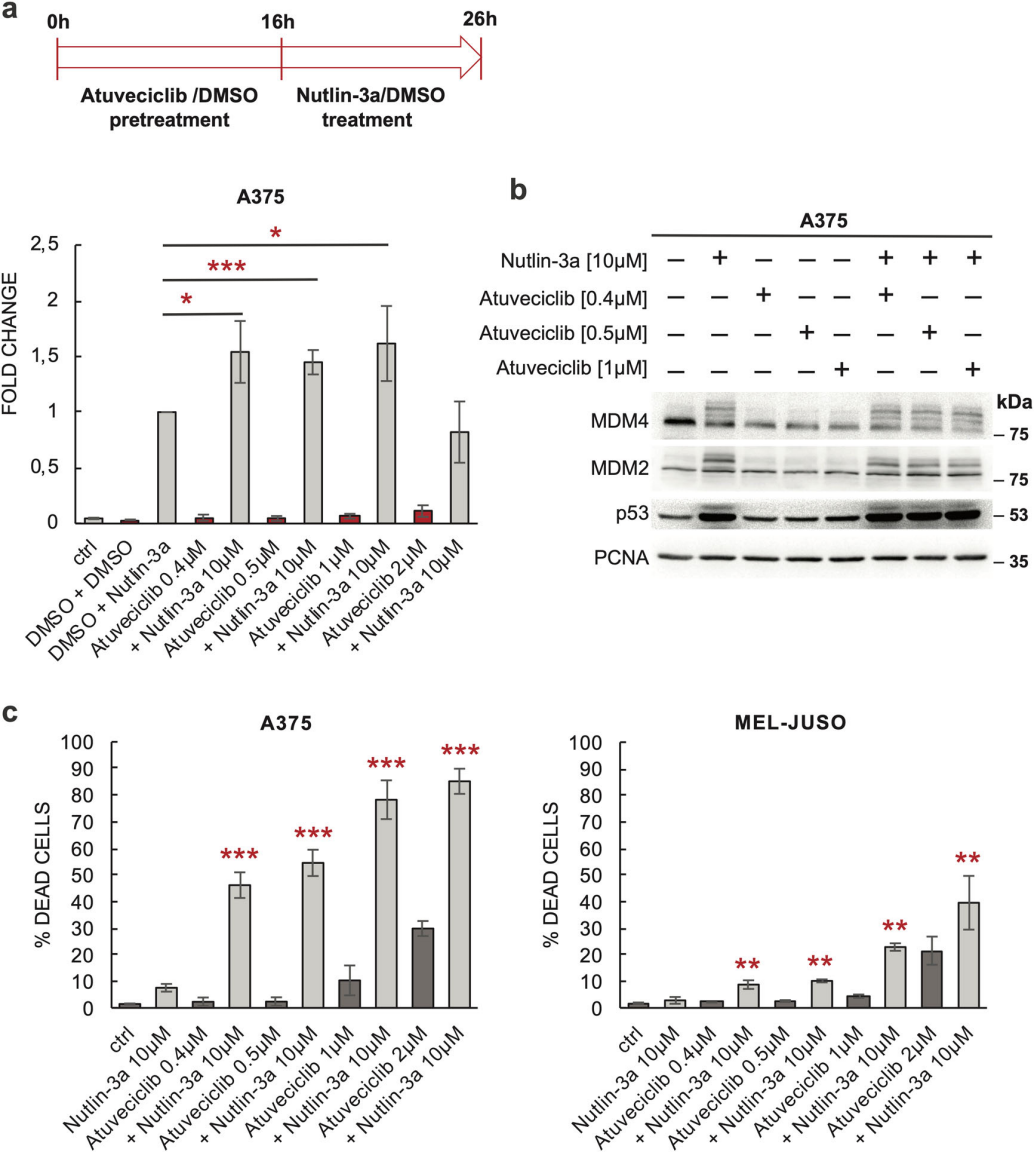
Atuveciclib acts synergistically with nutlin-3a in melanoma. **a** Activation of p53 by atuveciclib combined with nutlin-3a treatment. To avoid a complete transcriptional block and thus interference with the luciferase reporter system, A375 cells were pretreated with atuveciclib for 16 h, washed, and treated with nutlin-3a for 10 h. DMSO pretreatment and treatment was used for control = DMSO + DMSO; ctrl = untreated control. The values are relative to the DMSO + nutlin-3a sample and represent the mean ± SD; *N* = 3; *P < 0.05; ***P < 0.001. **b** Western blot analysis of cell lysates after 24-h co-treatment with atuveciclib and nutlin-3a. **c** Effect of the drug combination on melanoma cell viability. Propidium iodide viability assay was performed after a 48-h co-treatment with atuveciclib and nutlin-3a in A375 and MEL-JUSO cells. The values represent the mean ± SD; *N* = 3; **P < 0.01; ***P < 0.001.

As CDK9 inhibition preferentially targeted MDM4, we hypothesized that the CDK9 inhibitor atuveciclib might cooperate with nutlin-3a, a small-molecule inhibitor of MDM2-p53 interaction^49^, in stimulating p53 activity. A previous study reported that the CDK inhibitor roscovitine partly interfered with the expression of a p53 activity reporter in MCF7 cells and potent activation of p53-dependent luciferase expression several hours after roscovitine removal^19^. That is why we chose an experimental design in which cells were pretreated with CDKI, followed by its removal and treatment with the second drug. More specifically, A375 cells stably transfected with the p53 activity reporter construct pGL4.38[luc2P/p53RE/Hygro] were treated with increasing concentrations of atuveciclib for 16 h. Then the medium was replaced with a fresh medium with or without nutlin-3a. Cells were lysed 10 h later, and luciferase activity was determined. Results suggested that the 16-hour pre-treatment with atuveciclib significantly enhanced p53 activity induced by nutlin-3a (Fig. 5a).

Western blot analysis of A375 cells co-treated with atuveciclib and nutlin-3a for 24 h showed a decrease in MDM4 levels in atuveciclib-treated cells and accumulation of high molecular weight forms of MDM2 and MDM4 in nutlin-treated cells. Nutlin-3a also promoted substantial p53 accumulation, while the increase in p53 stability induced in this experiment by atuveciclib was comparatively small (Fig. 5b).

Inhibition of p53 function by MDM4 has been reported to be necessary for melanoma development and melanoma cell survival^24^. When we treated melanoma cells with the combinations of nutlin-3a and increasing concentrations of atuveciclib, in the propidium iodide-exclusion viability assay, we observed a powerful synergistic effect of the drug combination on killing A375 cells. Cooperation between nutlin-3a and atuveciclib in inducing cell death was also observed in MEL-JUSO melanoma cells (Fig. 5c).

### CDK9 controls MDM4 expression in human pluripotent stem cells

Our results suggested that CDK9 activity contributes to the control of p53 in tumor cells by maintaining high levels of MDM4. We wanted to test the possibility that continuous CDK9 activity could be required to maintain MDM4 expression also in non-cancer cell types.

In addition to its vital role in genomic stability and tumor suppression, p53 has also been implicated in stem cell biology and the control of cell differentiation^50^. In human embryonic stem cells (hESCs), p53 expression has to be strictly controlled to prevent apoptosis and differentiation^51^. Nutlin-3 can induce rapid hESCs differentiation, indicating that MDM2 contributes to the control of p53 levels, also in pluripotent stem cells^52^. In contrast, the role of MDM4 in the control of p53 in pluripotent cells has yet to be determined.

**Fig. 6:**
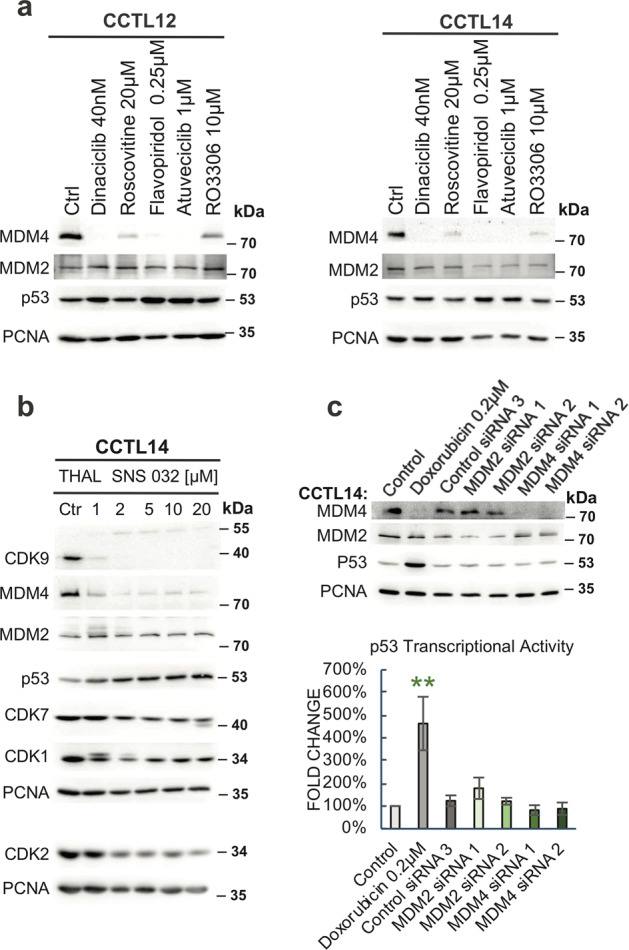
CDK9 controls MDM4 levels in human pluripotent stem cells. **a** CDK9 inhibition leads to MDM4 downregulation in hESCs. Western blot analysis of CCTL14 and CCTL12 cell lysates after 24-h treatment with selected CDK inhibitors. **b** Selective knockdown of CDK9 by THAL-SNS-032 PROTAC promotes MDM4 depletion in hESCs. Western blot analysis of CCTL14 lysates after 16-h THAL-SNS-032 treatment. **c** MDM4/MDM2 knockdown does not affect p53 levels in hESCs. Western blot and p53 transcriptional activity analysis in hESCs upon siRNA-mediated knockdown of MDM2 and MDM4. DNA-damaging agent doxorubicin served as a positive control. The p53 activity values are relative to control and error bars represent the SD; *N* = 3; double asterisk denotes *P* < 0.01.

We analyzed the effect of CDKIs on MDM4 expression in two different hESCs lines CCTL12 and CCTL14. As in cancer cells, MDM4 expression was strongly inhibited by dinaciclib, roscovitine, flavopiridol, atuveciclib, and RO3306 in both lines, while MDM2 levels changed only minimally (Fig. 6a). The CDK9 PROTAC THAL-SNS-032 depleted cellular levels of CDK9 (and partly also other CDKs) in hESCs already at 1 μM concentration (Fig. 6b), presumably due to higher expression of CRBN ubiquitin ligase in hESCs compared to A375 melanoma. CDK9 depletion was also accompanied by a nearly complete depletion of MDM4, while MDM2 levels remained unchanged (Fig. 6b). This data indicated that CDK9 activity also controls *MDM4* expression in hESCs.

Next, we used CCTL14 cells stably transfected with the reporter construct pGL4.38[luc2P/p53 RE/Hygro] to assess the impact of MDM4 modulation on p53 activity in hESCs. Cells were transiently transfected with siRNAs targeting *MDM2* and *MDM4* expression, and changes in p53 levels and activity were analyzed using western blotting and luciferase activity assays (Fig. 6c). Treatment with the DNA-damaging drug doxorubicin served as a positive control. The knockdown of MDM2 and MDM4 levels did not affect p53 protein levels and transcriptional activity, suggesting that the primary function of MDM2 and MDM4 proteins in human pluripotent stem cells may not be the control of p53 activity.

Interestingly, both MDM2 and MDM4 have been reported to have p53-independent functions in the regulation of DNA damage response and DNA repair^53^. In our experiments, the immunofluorescence analysis of MDM4 subcellular localization in hESCs showed cells in which MDM4 localized to discrete foci in the nucleus (Fig. S2a). We treated hESCs with DNA-damaging ionizing radiation and analyzed the subcellular localization of the MDM4 protein. The occurrence of MDM4 foci per nucleus (counted as total foci/ total nuclei), and the number of nuclei containing foci, increased significantly after the treatment (Supplementary Fig. S2a). The nuclear localization pattern of MDM4 foci was verified on maximum intensity projections of confocal image stacks (Supplementary Fig. S2b). These findings indicate that MDM4 might participate in the DNA damage response in pluripotent cells. More research will be necessary, however, to determine if the CDK9-dependent expression of MDM4 significantly contributes to the maintenance of genomic stability in hESCs and whether CDK9 inhibitors might disrupt DNA repair in pluripotent cells.

## Discussion

The ability of small-molecule CDKIs flavopiridol, olomoucine, and roscovitine to promote p53 accumulation in cancer cells was first reported more than twenty years ago^14,54^. In contrast to chemotherapy or radiation, activating p53 via DNA damage response, the stabilization and activation of p53 by CDKIs seemed to be mediated by a non-genotoxic mechanism. Therefore, the findings sparked considerable interest as a potential tool for p53 reactivation in tumors retaining wild-type p53^4,15,17^. However, the mechanism of p53 activation in response to CDKIs proved to be rather complicated. Reports indicated that the downregulation of MDM2 expression was responsible for p53 stabilization and activation^19,20^, while other reports suggested that MDM2 expression was not the primary CDKI target in the p53 pathway^21^. Already the early studies suggested that p53 activity might be stimulated by low doses of CDKIs, while higher doses of the drugs might lead to decreased MDM2 levels and p53 stabilization^55^. The observation that CDKIs caused nucleolar disruption and inhibition of the processing of ribosomal RNA suggested that p53 activation might constitute a response to nucleolar stress^14,56^. RPL5, RPL11, and other ribosomal proteins mediating the nucleolar stress response could inhibit MDM2 activity towards p53 even in the absence of changes in MDM2 levels^57^.

In a significant proportion of cancers, targeting MDM4 could lead to the reactivation of the p53 pathway^58^. Based on the analyses of *MDM4* gene amplification and overexpression on the mRNA level, the initial estimates of MDM4 overexpression in human cancers were around 17 %^59^. However, the availability of new specific and sensitive antibodies for MDM4 protein detection led to a realization that many tumors overexpress MDM4 protein without an apparent increase of *MDM4* mRNA, suggesting post-transcriptional regulation of MDM4 levels. In melanoma, more than 65 % of tumors express high MDM4 levels^24^. A switch in the alternative splicing of *MDM4* mRNA exon 6 was identified as the primary driver of MDM4 overproduction in human tumors^40^.

The current drug development strategies for targeting MDM4 involve mainly small molecules and stapled peptides designed to disrupt the physical interaction between MDM4 and p53, and inhibitors of MDM2-MDM4 heterodimerization^60^. Antisense oligonucleotides and specific inhibitors of ﻿Cdc-like kinases (CLKs) were shown to promote a switch in *MDM4* alternative splicing in cancer cells^40,61^. Clinically used fluoroquinolone antibiotics were recently reported to induce a switch in *MDM4* mRNA splicing, and benzimidazole anthelmintics inhibited MDM4 expression in melanoma cells^62,63^, indicating opportunities for drug repositioning.

In the current study, we identified CDK9 activity as critical for maintaining MDM4 levels. We show that CDK9 inhibition or knockdown can have a profound effect on the levels of MDM4 oncoprotein in cancer cells overexpressing *MDM4* and human pluripotent stem cells, with a minimal impact on *MDM2* expression. We found that CDK9 inhibitor atuveciclib synergizes with MDM2 inhibitor nutlin-3a in promoting p53 activity in melanoma cells. This synergy could also contribute to the previously reported sensitization of cancer cells to nutlin-3 by other CDKIs^64,65^.

In some experiments, we observed that the downregulation of MDM4 by CDKIs, especially at higher concentrations, led to p53 stabilization. While we cannot exclude the possibility that CDKIs inhibit the expression of other E3s for p53, this variability could also stem from the extreme complexity of the network of physical and functional interactions between MDM2, MDM4, and p53, including several feedback loops.

Both MDM2 and MDM4 can directly interact with the N-terminal domain of p53 and inhibit the transactivation of target genes^27,66,67^. MDM2 is a direct transcriptional target of p53 but can serve, in the form of MDM2/MDM2 homodimer, as a ubiquitin ligase for p53, targeting it for proteasomal degradation^68,69^. However, the MDM2 homodimer also serves as E3 for itself, limiting MDM2 stability and MDM2-mediated p53 degradation. In contrast, MDM4 cannot homodimerize and, on its own, lacks the E3 activity and cannot target p53 for degradation. That is why the downregulation of MDM4 does not need to induce direct changes in the p53 levels. However, MDM4 can also form heterodimers with MDM2 that act as E3 towards p53 and MDM4, targeting these two proteins for degradation, but not MDM2^29,70,71^. Therefore, at equimolar ratios, MDM4 can stabilize MDM2 and promote its activity towards p53.

In contrast, higher levels of MDM4 can outcompete MDM2 in binding p53, inhibiting p53 degradation, but the p53 protein remains transcriptionally inactive due to the interaction with MDM4. Moreover, MDM4 lacks the nuclear localization signal and, in the absence of MDM2, localizes predominantly in the cytoplasm. The nuclear localization signal of MDM2 can promote MDM4 translocation in the form of MDM2/MDM4 heterodimers into the nucleus where the majority of wild-type p53 protein resides^72^. This interconnection means that the immediate impact of MDM4 downregulation on p53 protein stability is highly dependent on the actual ratios between MDM2, MDM4, and p53.

It is of interest that MDM4 overexpression was shown to abrogate p53 activation in response to ribosomal stress and promote cancer cell resistance to low doses of 5-fluorouracil, which at low concentrations activates p53 by inducing ribosomal stress without significant DNA damage^73^. The strong effect of CDKIs on p53 activity in cancers overexpressing *MDM4* could, depending on the MDM2 and MDM4 protein ratios, simultaneously reflect the following mechanisms: (1) the disruption of MDM4-mediated inhibition of p53 transcriptional activity, (2) the change in the abundance of MDM2/MDM4 heterodimers vs. MDM2/MDM2 homodimers, and (3) the enhanced inhibition of MDM2 activity by ribosomal proteins in the absence of MDM4.

Numerous small-molecule CDK inhibitors underwent extensive preclinical testing, and many also entered clinical testing, including inhibitors targeting CDK9^5,35^. Our findings suggest that patients with tumors that overexpress MDM4 might benefit from targeting CDKs, which could stimulate the development of new therapies. One could also envisage a strategy that combines various approaches targeting MDM4 to ensure maximal therapeutic efficacy and prevent resistance to therapy.

## Materials and methods

### Cell culture, treatments, siRNA transfections

Human cell lines A375, MEL-JUSO, and MCF7 were purchased from the European Collection of Animal Cell Cultures (ECACC), Salisbury, UK, and from the German Collection of Microorganisms and Cell Cultures GmbH (DSMZ), Brunswick, Germany. Cells were maintained in RPMI-1640 medium (Sigma-Aldrich) supplemented with 10% fetal bovine serum, 2mM L-glutamine, and antibiotics penicillin (100 IU/ml) and streptomycin (100 μg/ml). Human embryonic stem cell lines CCTL12 and CCTL14 were obtained from the Centre for Cell Therapy and Tissue Repair, Charles University, Prague. hESCs were cultured on Matrigel (BD) in mouse embryonic fibroblast feeder-conditioned hES medium at 37 °C in a humidified atmosphere with 5% CO_2_. All cell lines were checked regularly for mycoplasma contamination.

The small-molecule compounds were purchased from Enzo Biochem (nutlin-3a), Sigma-Aldrich (doxorubicin, roscovitine, flavopiridol, DRB), Selleckchem (dinaciclib, AT7519, palbociclib, SNS-032), Tocris (THAL-SNS-032), Santa Cruz Biotechnology (RO3306, NU6102), and MedChemExpress (ribociclib, THZ1, THZ531, Senexin A, atuveciclib).

For the treatment of cells, the stock solutions in DMSO were diluted in pre-warmed cell culture medium and added to the cells. The range of concentrations active in human cells was selected according to the literature. Three different concentrations were used for each compound tested. Control cells were treated with the corresponding amount of vehicle (DMSO).

Gene knockdown by siRNA transfection was performed using the X-tremeGENE™ siRNA Transfection Reagent (Roche) according to the manufacturer’s instructions, and transfected cells were harvested 48 h later. Knockdown efficiency was verified by western blot analysis. The siRNAs used in this study are listed in the Supplemental Table S1.

### Western blot analysis

Cancer cell lysates in 2x Laemmli sample buffer were resolved by SDS-PAGE in 10% polyacrylamide gels, and proteins were transferred to PVDF membranes (Merck Millipore) using the Trans-Blot SD semi-dry transfer system (Bio-Rad). Membranes were blocked in 5% milk in TBS-Tween for 50 min at room temperature and incubated overnight at 4 °C with primary antibodies. After a wash in 1% dry milk in TBS-Tween for 4 × 7 min, the membranes were incubated for 1 hour at room temperature with the horseradish peroxidase-conjugated anti-rabbit or anti-mouse secondary antibodies (Santa Cruz Biotechnology). Proteins of interest were visualized using the ECL substrate (Thermo Scientific) or Immobilon Western Chemiluminescent HRP Substrate (Merck Millipore) in the G:BOX detection system (Syngene). hESCs samples were lysed in 1% SDS (pH 6.8), and protein concentration in the lysates was determined using the DC protein assay kit (Bio-Rad). Equal amounts of protein were loaded in all wells.

### EdU incorporation assay

Cell proliferation was assessed using the EdU Flow Cytometry Kit 488 (BCK-FC488, Merck Millipore) following the manufacturer’s instructions. Briefly, A375 cells were seeded at a concentration of 30,000 cells/ml in 12-well plates and left to adhere overnight. After a subsequent 24-h treatment with palbociclib and pan-CDK inhibitors dinaciclib and flavopiridol, the cells were incubated with a thymidine nucleoside analog 5-ethynyl-2′-deoxyuridine (EdU) for 2 h, harvested and washed in 1% BSA in PBS. In the next step, the cells were fixed with 100 μl of 4% paraformaldehyde in PBS for 15 min at room temperature, washed again in 1% BSA in PBS, and permeabilized in 100 μl of 1x saponin-based buffer for 20 min. For the click chemistry reaction, 500 μl of freshly prepared assay cocktail was added to each sample for 30 min, protected from light. Cells were washed in 3 ml of 1x saponin-based buffer and resuspended in 300 μl 1x saponin-based buffer. The fluorescent signal generated by proliferating EdU-positive, 6-FAM-azide-labeled cells was detected by flow cytometry using the Beckman Coulter 426 Cytomics FC 500 flow cytometer at 496 and 516 nm emission wavelength.

### Antibodies

Western blots were probed with anti-MDM4 mouse mAb (8C6; Merck-Millipore), anti-cdc2 mouse mAb (POH1; Cell Signaling Technology), anti-CDK2 rabbit mAb (78B2; Cell Signaling Technology), anti-CDK7 mouse mAb (MO1; Cell Signaling Technology), anti-CDK9 mouse mAb (D-7, Santa Cruz Biotechnology) and anti-Mcl-1 rabbit pAb (M8434, Sigma-Aldrich). Anti-MDM2 mouse monoclonal antibodies 2A9 and 2A10, anti-PCNA mouse mAb PC-10, anti-p21 mouse mAb 118, and anti-p53 mouse mAb DO-1 were generously provided by Dr. Bořivoj Vojtěšek (Masaryk Memorial Cancer Institute, Brno, Czech Republic).

### p53 transcriptional activity analysis

Wild-type p53 melanoma cell lines A375 and MEL-JUSO and the hESC cell line CCTL14 were stably transfected with the p53-responsive luciferase reporter construct pGL4.38 [luc2P/p53 RE/Hygro] (Promega). For determining firefly luciferase activity, drug-treated and control DMSO-treated cells were lysed on ice in the Reporter Lysis Buffer (Promega) for 15 min, and protein concentrations in the lysates were determined using the Protein Assay Dye Reagent Concentrate (Bio-Rad). The luminescence was measured using the Luciferase Assay System (Promega) in the TriStar² LB 942 reader (Berthold Technologies Bad Wildbad, Germany). To calculate relative p53 transcriptional activity, the luminescence values were normalized to protein concentrations and the activity obtained in control cells.

### Colony formation assay

The ‘plating before treatment’ setup was used. Post-treatment, cells were washed twice with pre-warmed PBS and further cultured in the fresh medium for approximately two weeks, until the formation of visible colonies. Detached cells were washed, returned to the dishes, and further cultured as well. For staining, cells were washed thoroughly with PBS, and a mixture of 6.0% glutaraldehyde and 0.5% crystal violet was added for one hour. In the last step, the dishes were rinsed with tap water and left to dry at room temperature.

### Immunofluorescence microscopy

Cells on coverslips were fixed with 4% paraformaldehyde in phosphate-buffered saline (PBS) and blocked with 1% BSA (Sigma-Aldrich) plus 0.1% Triton X‐100 (Sigma-Aldrich) in PBS. Mouse monoclonal anti-MDM4 (clone 8C6, Merck-Millipore) was applied at 1:200 dilution in PBS-Tween overnight at 4 °C. Next, the coverslips were washed in PBS and incubated for 1 h with Alexa 488-conjugated anti-mouse secondary antibody (Thermo Fisher Scientific) diluted 1:500 in PBS-Tween. Cell nuclei were counterstained with 4′,6-diamidine-2′-phenylindole dihydrochloride (DAPI) (Sigma-Aldrich), and fluorescence microscopy was performed using the LSM700 confocal microscope (Carl Zeiss). To analyze the MDM4 foci, the number of foci formed inside the nuclei, as well as the total number of nuclei in the picture, was counted manually in 10 representative snapshots of each sample. The foci/nuclei ratio was calculated as total foci inside nuclei / total nuclei. The number of foci-containing nuclei per snapshot was also assessed. The nuclear area was analyzed on maximum intensity projections (MIP) of confocal z-stacks using the Zen 2.3. imaging software (Zeiss).

### Propidium iodide viability assay

Cells were collected 48 h post-treatment by trypsinization and washed once in PBS. Cell pellets were resuspended in ice-cold PBS, and 1 μg/ml Propidium iodide (PI) (Sigma-Aldrich) was added to the suspension. The fraction of dead (PI-positive) cells was identified using the Beckman Coulter Cytomics FC 500 flow cytometer at 620 nm emission wavelength.

### Real-time reverse transcription PCR (qRT-PCR)

Total RNA was isolated using the RNA Blue reagent (Top-Bio) according to the manufacturer’s instructions, and RNA concentration and purity were assessed using NanoDrop 1000 (Thermo Scientific). In all, 1 μg of RNA was transcribed into cDNA in the total volume of 20 μl using oligo-(dT)15 primer and Transcriptor Reverse Transcriptase (Roche). Quantitative RT-PCR analysis was performed using the LightCycler 480 Instrument (Roche). The final reaction volume of 10 μl included 1 μl of cDNA template, 5 μl of 2x TaqMan Gene Expression Master Mix (Applied Biosystems), 0.5 μl of the respective primer/probe set labeled with FAM/MGB and 0.5 μl of primer/probe set labeled with VIC/MGB (for GAPDH) and 3 μl of water. The following TaqMan Gene Expression Assays were used: MDM4/MDMX (Hs00967238_m1), BB3/PUMA (Hs00248075_m1), and CDKN1A/p21 (Hs00355782_m1), all from Thermo Fisher Scientific. PCR amplification of GAPDH (#4310884E, Thermo Fisher Scientific) served as a loading control. The PCR reaction was carried out with dual-color hydrolysis probe/UPL probe as follows: 10 min pre-incubation at 95 °C and 45 cycles of amplification: 10 s at 95 °C, 30 s at 60 °C and 1 s at 72 °C. Reactions were performed in triplicates, and the mean Ct value of each triplicate was used for analysis by the 2(- Delta Delta Ct) method.

### mNET-Seq data analysis

The mNET-Seq data from Gressel et al.^48^ were downloaded via the ENA database under accession number GSE96056 in a raw fastq format. A quality check of raw reads was carried out by FastQC^74^. The adapters and quality trimming of raw fastq reads was performed using Trimmomatic v. 0.36^75^ with settings CROP:250 LEADING:3 TRAILING:3 SLIDINGWINDOW:4:5 MINLEN:35. Trimmed mNET-Seq reads were mapped against the human genome (hg38) and Ensembl GRCh38 v.94 annotation using STAR v. 2.5.3a^76^ as splice-aware short read aligner and default parameters except –outFilterMismatchNoverLmax 0.1 and –twopassMode Basic. Resulting alignment files were further filtered using samtools v. 1.4.1^77^ software. Only reads mapped in pair remained in the data set, reads with mapping quality below 7, and all PCR and optical duplicates were filtered out. Filtered alignment files were used to produce coverage bedgraph files per each sample via deepTools bamCoverage v. 3.3.0^78^. Bedgraph files were further postproccessed to generate per position bedgraphs, and coverage counts were normalized by the number of mapped reads. Each condition consisted of three replicates, so the coverage within replicates in each condition was averaged to create one bedgraph per condition. Final bedgraph files were employed to generate Fig. 4b using R programming language, especially its libraries data.table, reshape2, and ggplot2.

### Statistical analysis

A minimum of three biological replicates of each experiment comparing experimental versus control conditions was performed. The exact number of independent experiments is indicated in the figure legends. *P* values of *P < 0.05, **P < 0.01, and ***P < 0.001 were considered statistically significant. All the data obtained were tested for normal distribution and homogeneity of variance before further analysis. Statistical significance was evaluated using two-tailed Student’s *t*-tests or one-way analysis of variance (ANOVA) using the GraphPad Prism 8 software (GraphPad Software, San Diego, USA). Data are presented as mean ± SD. Average values and SDs were calculated using Microsoft Excel.

## Supplementary information

Supplementary Figure Legends

Figure S1

Figure S2

Table S1
