# Supplementary material for: CDK9 activity is critical for maintaining MDM4 overexpression in tumor cells
Source: Cell Death Dis. 2020 Sep 15;11(9):754. doi: 10.1038/s41419-020-02971-3 (PMC7494941; doi:10.1038/s41419-020-02971-3)
Supplement: Supplementary file 1 — Supplementary Figure Legends [file 41419_2020_2971_MOESM1_ESM.docx]

**Supplementary Information**

**Supplementary Figure Legends**

**Figure S1**. **Effect of selected CDK inhibitors on cell cycle progression. Effect of RO3306 and THZ531 on MDM4 levels. Effect of MDM4 knockdown on p53 activity in A375 cells. (a)** CDK inhibitors dinaciclib, flavopiridol, and palbociclib suppress A375 melanoma cell cycle progression in sub-micromolar concentrations used in this study. The proportion of EdU-positive cells determined by flow cytometry in A375 cells exposed to different concentrations of CDK inhibitors for 24 h. The values represent the mean ± SD; N = 3; ***: P < 0.001. **(b)** CDK1 inhibitor RO3306 and CDK12/13 inhibitor THZ531 downregulate MDM4 levels in melanoma cells. Western blot analysis of A375 cell lysates after 24-hour treatment with the indicated concentrations of the compounds. Data from three independent experiments are presented. **(c)** MDM4 knockdown activates p53 in A375 melanoma cells. A375 cells bearing the pGL4.38[luc2P/p53 RE/Hygro] p53 activity luciferase reporter construct were transiently transfected with siRNAs targeting MDM4 transcripts. Relative p53 transcriptional activity was determined, and the efficacy of MDM4 knockdown was verified by western blot analysis. GFP-targeting siRNAs (control siRNA 2 and 3) were used as controls, and 24-hour treatment with the CDK9 inhibitor atuveciclib was used for reference. Data from three independent experiments are shown, the values in the graph represent the mean ± SD; N = 3; *: P < 0.05.

**Figure S2.** **MDM4 localizes to distinct foci within nuclei of hESCs. (a)** Ionizing radiation promotes MDM4 foci formation. Immunofluorescence images of MDM4 and DAPI co-localization immediately (0h) and 10h after irradiation, ten representative snapshots of each sample were taken. The number of nuclear MDM4 foci and the number of cell nuclei per snapshot were determined, and the Foci/Nuclei ratio was calculated. The changes in the number of nuclei containing foci 0 hours and 10 hours after irradiation were also determined. The values represent the mean ± SD; N = 3; **: P < 0.01; ***: P < 0.001. **(b)** 3D reconstruction of the nuclear area of a representative CCTL14 cell containing MDM4 foci. Three maximum intensity projections (MIP) of confocal z-stacks in the ‘Ortho view’ display mode are presented.
