# Supplementary figures and images for: CDK9 activity is critical for maintaining MDM4 overexpression in tumor cells
Source: Cell Death Dis. 2020 Sep 15;11(9):754. doi: 10.1038/s41419-020-02971-3 (PMC7494941; doi:10.1038/s41419-020-02971-3)

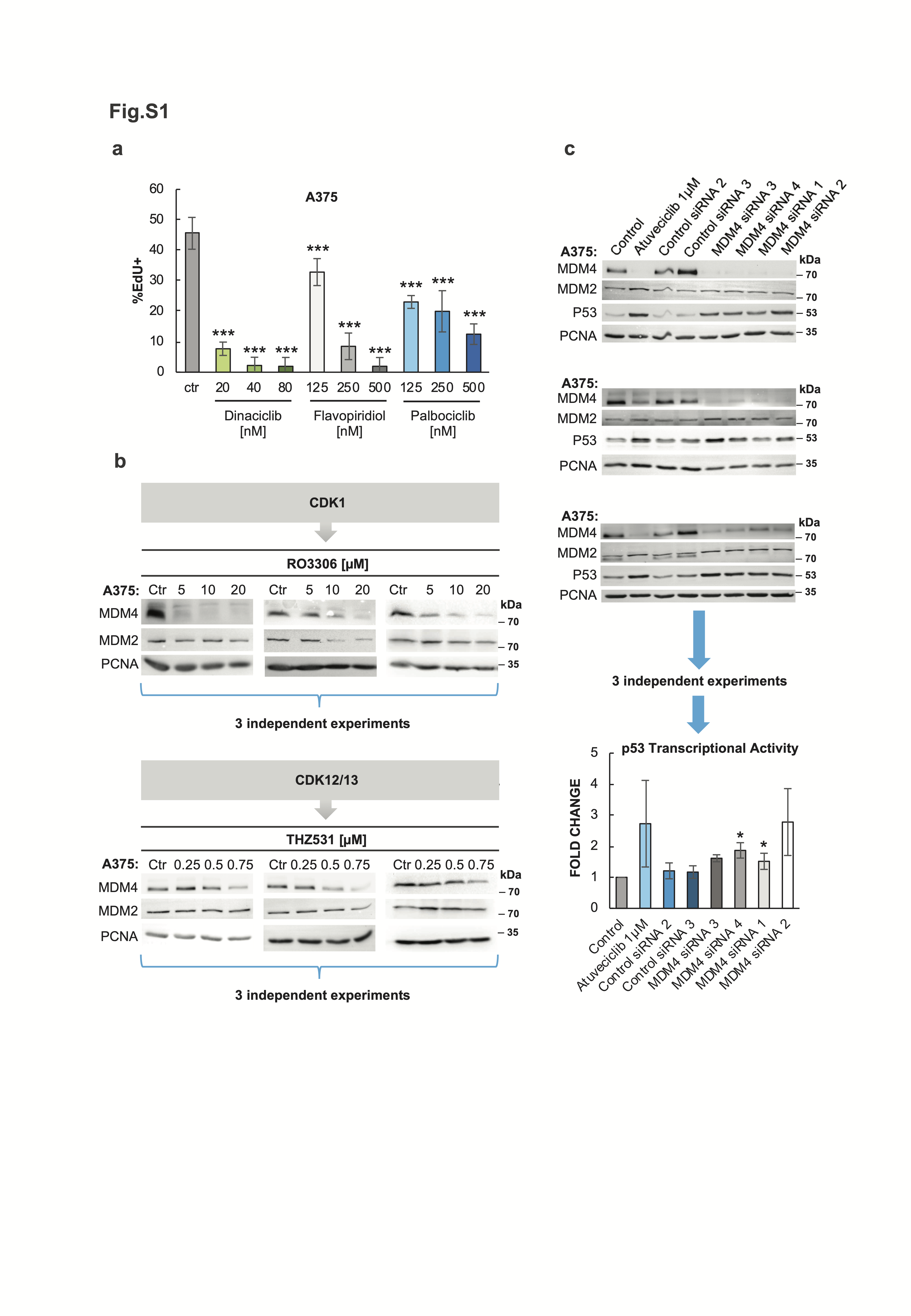

Supplement: Supplementary file 2 — Figure S1 [file 41419_2020_2971_MOESM2_ESM.tif]

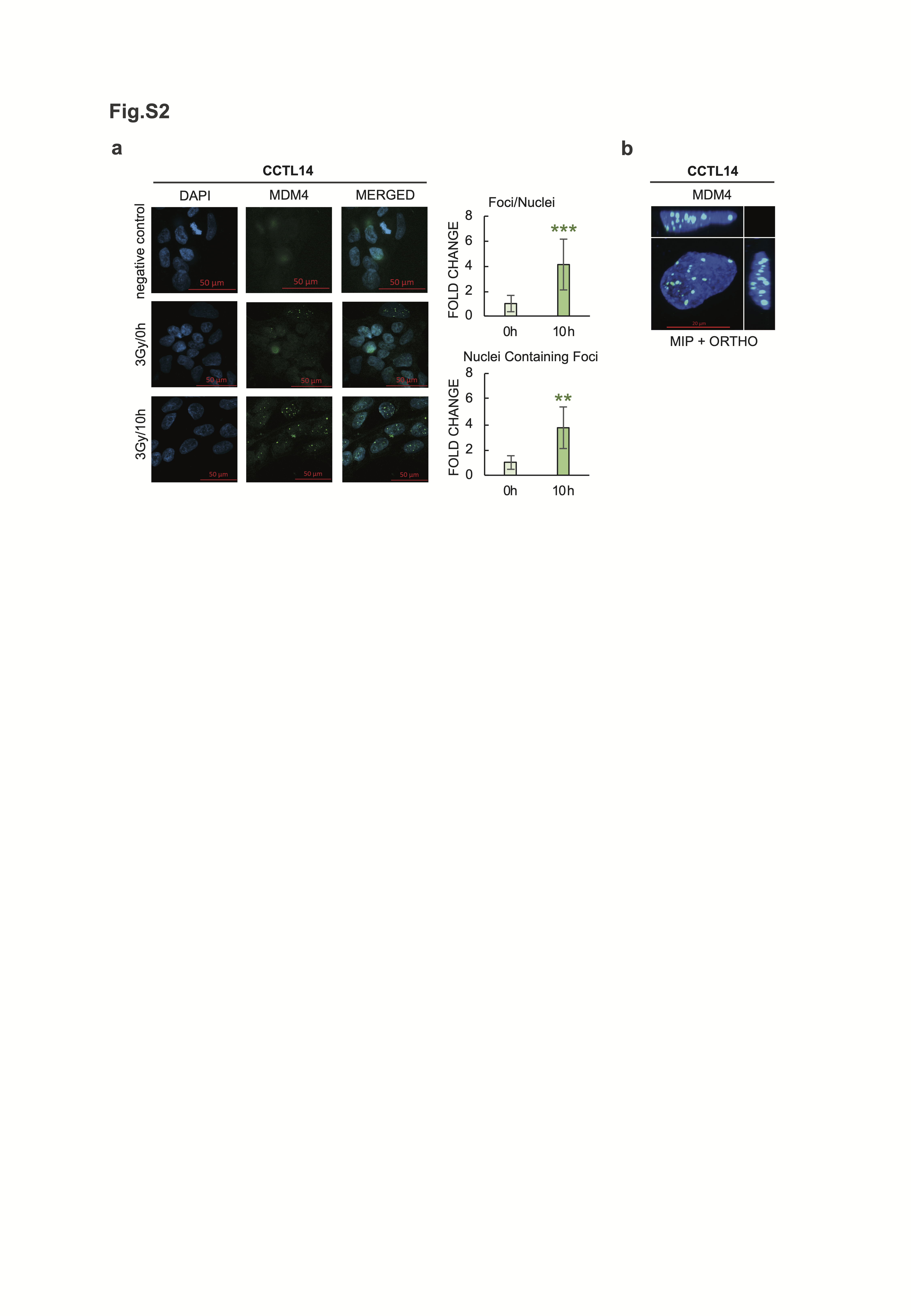

Supplement: Supplementary file 3 — Figure S2 [file 41419_2020_2971_MOESM3_ESM.tif]
