# Supplementary material for: CDK9 activity is critical for maintaining MDM4 overexpression in tumor cells
Source: Cell Death Dis. 2020 Sep 15;11(9):754. doi: 10.1038/s41419-020-02971-3 (PMC7494941; doi:10.1038/s41419-020-02971-3)
Supplement: Supplementary file 4 — Table S1 [file 41419_2020_2971_MOESM4_ESM.docx]

**Supplementary Table S1: si-RNA**

| CDK9 siRNA 1 | MISSION® esiRNA human CDK9 EHU053481 (Sigma-Aldrich) | |
| --- | --- | --- |
| CDK9 siRNA 2 | MISSION® Human Kinase CDK9 SIHK0371 (Sigma-Aldrich) | |
| CDK9 siRNA 3 | MISSION® Human Kinase CDK9 SIHK0372 (Sigma-Aldrich) | |
| CDK9 siRNA 4 | MISSION® Human Kinase CDK9 SIHK0373 (Sigma-Aldrich) | |
| Control siRNA 1 | AllStars Negative Control siRNA SI03650318 (Qiagen) | |
|  | |  |
| **Sequence (Sense strand)** | | **Note** |
| Control siRNA 2 | 5'-CUGGAGUUGUCCCAAUU-3' | Target: GFP |
| Control siRNA 3 | 5'-AGAAUUGGGACAACUCC-3' | Target: GFP |
| MDM2 siRNA 1 | 5'-GCCACAAAUCUGAUAGUAU-3' | (Maguire et al., 2008)* |
| MDM2 siRNA 2 | 5’-AGGAAUUUAGACAACCUGAA-3’ |  |
| MDM4 siRNA 1 | 5'-AUGCAUACAUUCUAGAGAATT-3' |  |
| MDM4 siRNA 2 | 5'-GGAAGGAUUGGUAUUCAGATT-3' |  |
| MDM4 siRNA 3 | 5'-GAACUACAGAAGACGAUAU(dTdT)-3' |  |
| MDM4 siRNA 4 | 5'-CCACGAGACGGGAACAUUA(dTdT)-3' |  |
|  |  |  |
| * | Maguire M, Nield PC, Devling T, Jenkins RE, Park BK, Polanski R, et al. MDM2 Regulates Dihydrofolate Reductase Activity through Monoubiquitination. Cancer Res. 2008 May 1;68(9):3232–42. | |
